# Dalbinol, a rotenoid from *Amorpha fruticosa L.*, exerts anti-proliferative activity by facilitating β-catenin degradation in hepatocellular carcinoma cells

**DOI:** 10.18632/oncotarget.17766

**Published:** 2017-05-10

**Authors:** Xiaohui Zhu, Xin Wu, Jing Cheng, Hongbo Liao, Xiaoqing Di, Lili Li, Rong Li, Yanfang Zhou, Xiangning Zhang

**Affiliations:** ^1^ Department of Pathophysiology, Guangdong Medical University, Zhanjiang 524023, Guangdong, China; ^2^ Guangdong Key Laboratory for Research and Development of Natural Drugs, Guangdong Medical University, Zhanjiang 524023, Guangdong, China; ^3^ Department of Laboratory Medicine, The First Affiliated Hospital of Sun Yat-sen University, Guangzhou 510080, Guangdong, China; ^4^ Department of Pathology, The Affiliated Hospital of Guangdong Medical University, Zhanjiang 524001, Guangdong, China

**Keywords:** dalbinol, hepatocellular carcinoma, Wnt/β-catenin, ubiquitin-proteasome degradation, cell proliferation

## Abstract

Hepatocellular carcinoma (HCC) is a highly malignant tumor, and the main cause of treatment failure is malignant proliferation. Aberrations in Wnt/β-catenin signaling are associated with HCC development. Despite the improvements in overall survival made over the past decade from the advent of molecularly targeted therapies, these treatments do not have efficacy in all patients with different pathogeneses. Therefore, there is a demand for novel chemotherapeutic agents for HCC. To this end, we built a natural compound library and screened out a rotenoid named dalbinol from the seeds of *Amorpha fruticosa L*. Our data demonstrated that dalbinol inhibited the growth of HepG2, HepG2/ADM and Huh7 cells in a concentration-dependent manner. Pharmacological experiments also showed that dalbinol suppressed growth and induced apoptosis in these HCC cell lines *in vitro*. Furthermore, we found that dalbinol promoted β-catenin degradation, which was mediated by the ubiquitin-proteasome pathway. To summarize, our results illustrate that dalbinol inhibited HCC cell growth by facilitating β-catenin degradation through the ubiquitin-proteasome pathway. Hence, we propose that dalbinol will be a promising agent for the treatment of HCC subtypes with aberrant Wnt/β-catenin pathway activation.

## INTRODUCTION

Hepatocellular carcinoma (HCC) is a leading cause of cancer-related deaths worldwide. It ranks 6th in terms of global incidence, and is steadily increasing in Western countries. The rise in the incidence of HCC is caused by the recent increase in hepatitis C virus (HCV) infection, a subtype of the hepatitis virus that is associated with human cancers [1]. Although incidence is low in the United States, where it is not among the cancers with the highest rate of occurrence [2], globally, HCC is the leading cause of death among cirrhotic patients, and the 3rd leading cause of cancer-related mortality, accounting for 600,000 deaths per year [3, 4].

Aberrations in the canonical Wnt signaling pathway contribute to congenital malformations, osteoporosis and cancers [5–7], including hepatocellular carcinoma [8–10]. β-catenin, as a transcription co-activator, is a critical component of the Wnt pathway and plays evolutionarily conserved roles in embryonic development and organismal growth from *Drosophila* to mammals. In its quiescent state, low β-catenin levels are maintained through an ubiquitin-proteasome degradation pathway following adenomatous polyposis coli (APC)-mediated transportation from the nucleus to the cytoplasm [11]. In malignancies, β-catenin accumulates in the nucleus primarily due to mutations in Wnt signaling components, such as APC [12], β-catenin [13] and Axin [14]. In fact, the stability and nuclear retention of β-catenin is beyond APC regulation in many cancer cells [15, 16]. β-catenin activity is downregulated by phosphorylation at several residues, which leads to its degradation. Phosphorylation at serines 33 and 37 creates a binding site for the E3 ubiquitin ligase β-TrCP, leading to β-catenin degradation [17].

HCC diagnoses are clinically confirmed only at advanced stages, meaning that in most cases, surgical resection is not suitable [18, 19]. As an alternative course, molecularly targeted therapies have improved patient survival. The most effective therapeutic agent has been sorafenib [20, 21], which targets BRAF, RAS, VEGFR and PDGFR. Following sorafenib, numerous agents have entered Phase II and III trials including sunitinib [22–24] (VEGFR, PDGFR and c-Kit), erlotinib [25, 26] (EGF), and everolimus [27] and Rapamycin [28] (mTOR inhibitors). Despite the great improvements, the available drugs are not efficacious in all HCC patients, especially those with different aberrations, such as Wnt/β-catenin pathway hyper-activation [27].

In recent years, many rotenoid derivatives have been described as promising anti-tumor agents. Among these, deguelin has attracted the most interest. Deguelin not only upregulates IGFBP3 [29] and represses c-Met/EGFR [30] signaling in breast cancer cells, but also induces apoptosis and inhibits proliferation in head and neck squamous carcinoma cells [31]. Another rotenoid, boeravinone, reverses drug resistance by blocking BCRP/ABCG2-triggered drug efflux in breast cancer cells [32], and 6-deoxyclitoriacetal also showed excellent anti-tumor activity in SW620, KATO, ChaGo, BT474 and HepG2 cells [33]. Dalbinol, a rotenone structural analogue, can be obtained from dried *Amorpha fruticosa L*. seeds [34]. In spite of its inhibiting effects on biomembrane formation in *Pseudomonas aeruginosa* [35], dalbinol, as an anti-tumor agent, has not yet been reported.

In this study, we extracted and purified dalbinol from dried *Amorpha fruticosa L*. seeds, and discovered its anti-cancer effects on HepG2, HepG2/ADM and Huh7 cells. Our results revealed dalbinol-induced apoptosis in HCC cell lines. What is more, it inhibited HCC cell growth by promoting the ubiquitin–proteasome-mediated degradation of β-catenin. Thus, dalbinol may be a promising agent for the treatment of HCC and other cancers with aberrant Wnt/β-catenin signaling.

## RESULTS

### Cytotoxic effects of dalbinol for normal hepatic cells and growth inhibition for HCC cells

**Figure 1:**
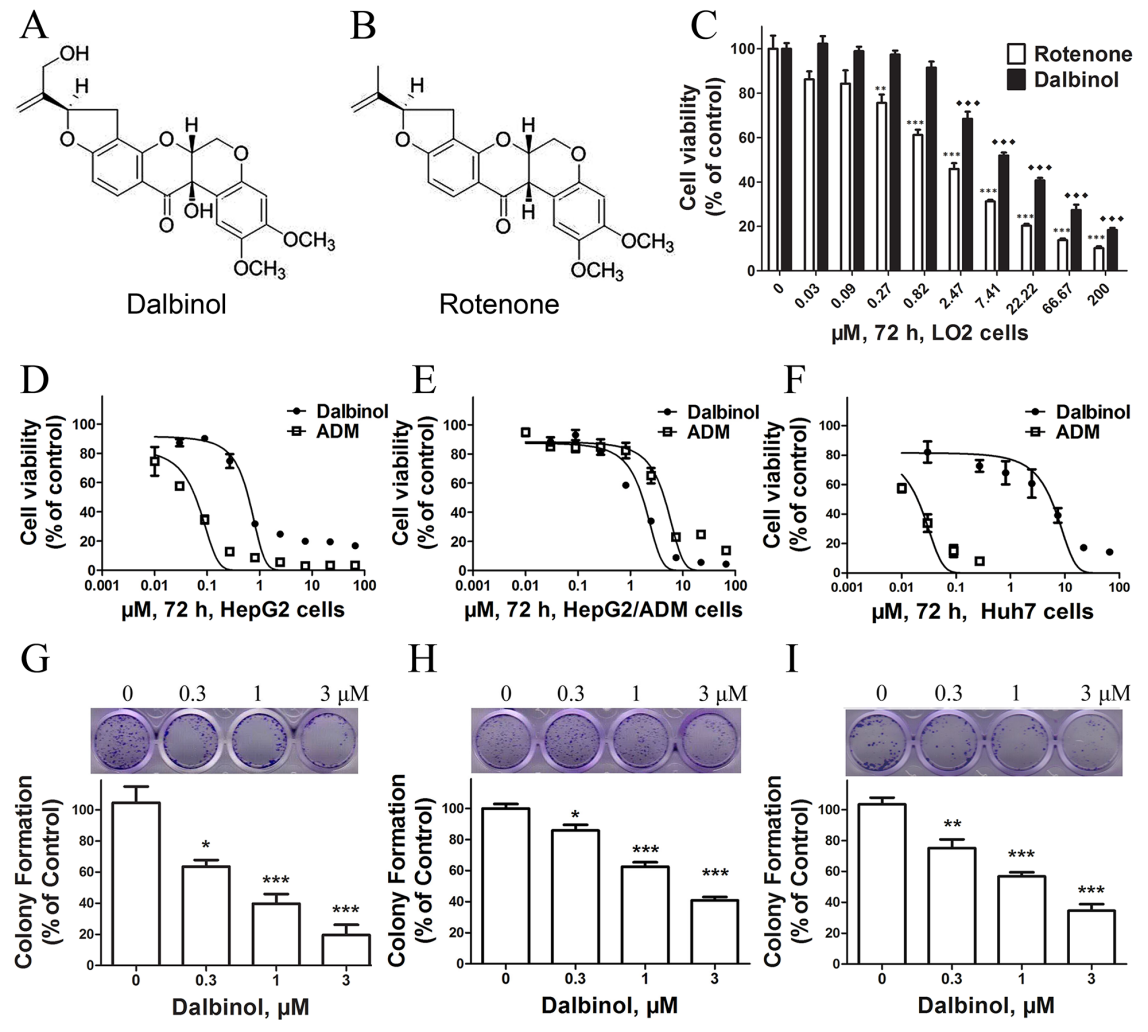
Chemical structures and cytotoxic effects of compounds on normal hepatic cells and HCC cell lines **(A, B)** The chemical structures of dalbinol (A) and rotenone (B). **(C)** Cytotoxic effects of dalbinol and rotenone on the normal hepatic cell line LO2. **(D, E, F)** The viability of HepG2 (D), HepG2/ADM (E) and Huh7 (F) cells treated with the indicated concentrations of dalbinol and Adriamycin for 72 h. **(G, H, I)** Representative images and statistical analysis from colony-forming assays using HepG2 (G), HepG2/ADM (H) and Huh7 (I) cells. All data are presented as means ± SD of three independent experiments. *P < 0.05, **P < 0.01, ***P < 0.0001, ♦P < 0.05,♦♦P < 0.01,♦♦♦P < 0.0001, compared with untreated cells.

The natural compound dalbinol (Figure 1A) is obtained from *Amorpha fruticosa L*. seeds and has a similar chemical structure to rotenone (Figure 1B). To evaluate the cytotoxic effects of dalbinol, the Cell Counting Kit-8 (CCK-8) assay was performed after exposing cells to dalbinol or rotenone for 68 h. The results showed that the IC_50_ values for dalbinol and rotenone for the normal hepatic cell line LO2 were 16.8 μM and 3.92 μM, respectively. Thus, dalbinol was less cytotoxic to LO2 cells than rotenone (Figure 1C).

We then examined the effects of dalbinol on the growth of the HCC cell lines HepG2 and HepG2/ADM. Cell viability was measured by CCK-8 assay after treating cells with increasing concentrations of dalbinol for 72 h. Equal volumes of DMSO (<0.1%) were added to cells as a control. Dalbinol inhibited the growth of HepG2 (Figure 1D), HepG2/ADM (Figure 1E) and Huh7 (Figure 1F) cells in a concentration-dependent manner. The IC_50_ values for dalbinol in HepG2, HepG2/ADM and Huh7 cells were 0.6 μM, 1.7 μM, and 5.5 μM, respectively, while the IC_50_ of Adriamycin for these same cells were 0.06 μM, 4.4 μM and 0.02 μM. To evaluate the effect of dalbinol on the long-term proliferation of HCC cells, the colony formation assay was performed. These results showed that dalbinol effectively inhibited colony formation in HepG2 (Figure 1G), HepG2/ADM (Figure 1H) and Huh7 (Figure 1I) cells.

### Apoptosis induction following dalbinol treatment

**Figure 2:**
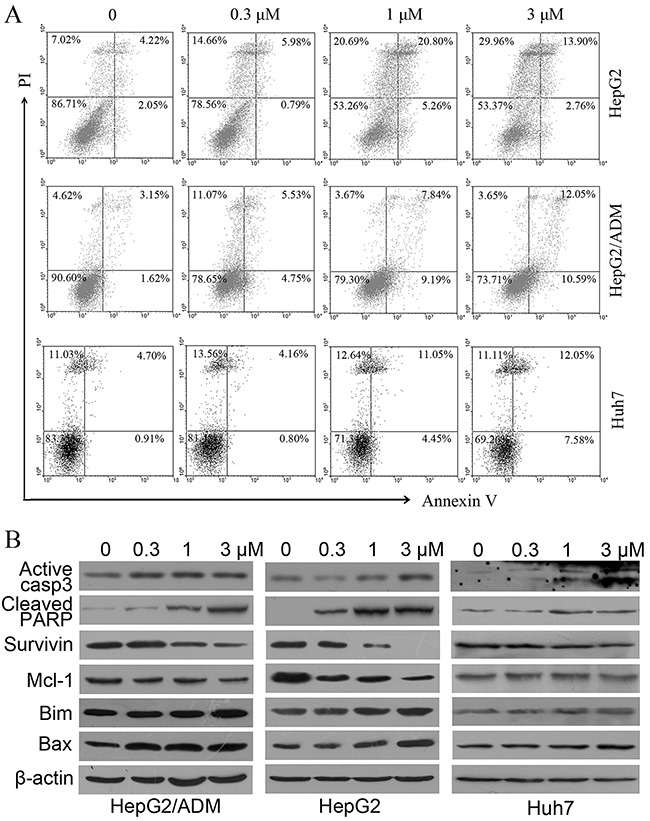
Dalbinol induced apoptosis in HepG2, HepG2/ADM and Huh7 cells **(A)** Cells were treated with increasing concentrations of dalbinol for 24 h, and apoptotic cells were analyzed by flow cytometry after Annexin V/PI staining. **(B)** Western blot analysis of apoptosis related proteins in HepG2, HepG2/ADM and Huh7 cells treated with the indicated concentrations of dalbinol for 24 h.

To evaluate whether dalbinol induced apoptosis, we conducted flow cytometry assay after Annexin V/propidium iodide double staining of dalbinol-challenged HCC cells. These data suggested a trend towards apoptosis induction in HepG2, HepG2/ADM and Huh7 cells in a concentration-dependent manner after dalbinol treatment, however the values were not significantly different (Figure 2A). We next investigated the levels of apoptosis-related proteins. These data demonstrated that dalbinol, in a concentration-dependent manner, induced an elevation of activated caspase-3 and cleaved PARP in HepG2, HepG2/ADM and Huh7 cells (Figure 2B). To further elucidate the mechanism of apoptosis induction, we detected the effects of dalbinol on members of the Bcl-2 family. As shown in Figure 2B, dalbinol reduced the level of the anti-apoptotic protein Mcl-1, and elevated the level of the pro-apoptotic proteins Bax and Bim.

### Dalbinol suppressed Wnt/β-catenin signaling in HCC cells

**Figure 3:**
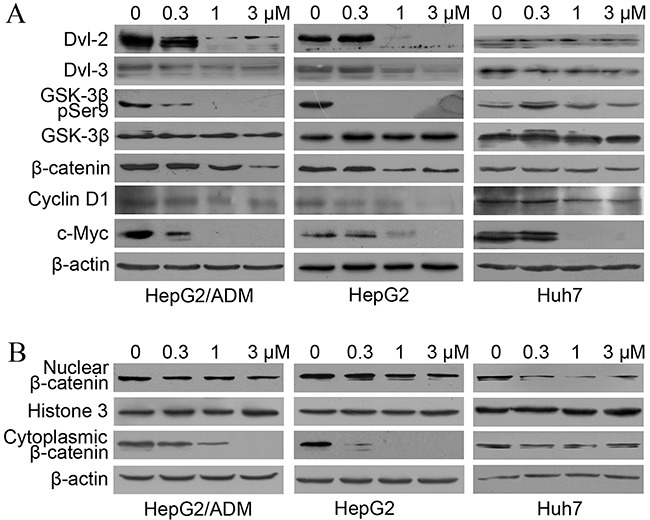

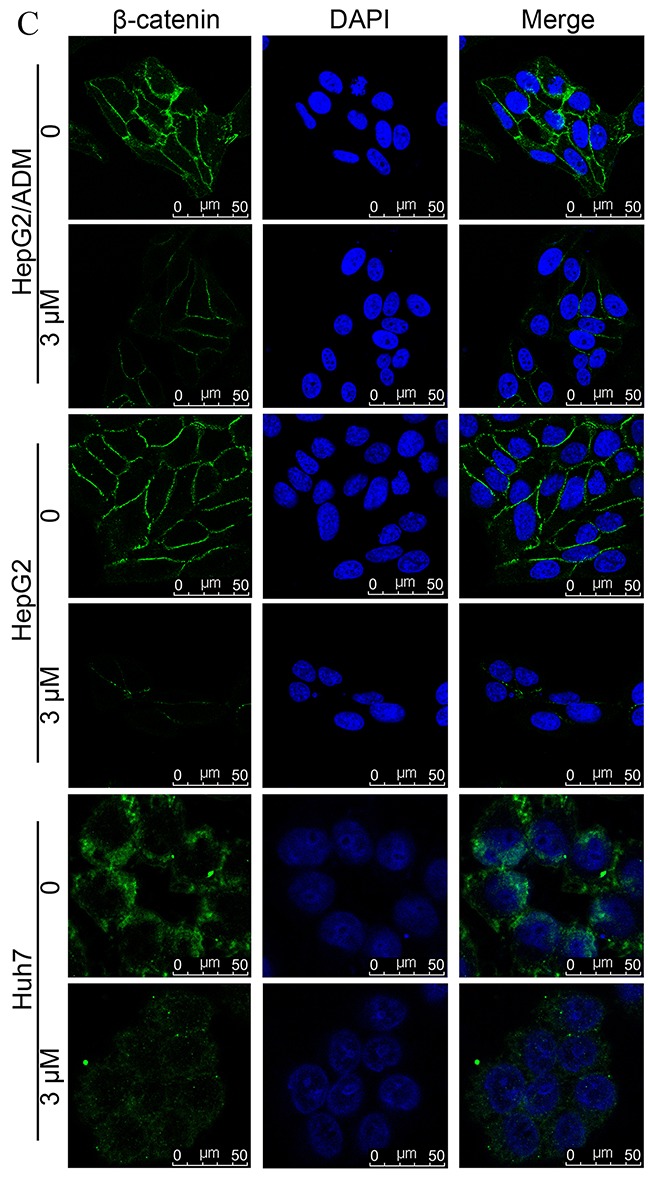
Dalbinol suppressed Wnt/β-catenin signaling in HCC cells **(A)** Western blot analyses of Wnt/β-catenin signaling components and its targets in HepG2, HepG2/ADM and Huh7 cells treated with the indicated concentrations of dalbinol for 24 h. **(B)** Cytoplasmic and nuclear β-catenin levels were analyzed by western blotting in HepG2, HepG2/ADM and Huh7 cells treated with dalbinol for 24 h. **(C)** Dalbinol reduced β-catenin level in HepG2, HepG2/ADM and Huh7 cells as evidenced by immunofluorescence staining.

The CCK-8 assays described above demonstrated that dalbinol inhibited cell proliferation in HCC cell lines, but the underlying mechanisms remained to be elucidated. It has been reported that deguelin, another rotenoid, suppresses growth of cancer cells by inhibiting Wnt signaling [36, 37]. Therefore, we hypothesized that dalbinol may also exert its anti-cancer activity by inhibiting Wnt signaling. To test this, we first examined β-catenin protein levels. To our surprise, total β-catenin levels were significantly reduced by dalbinol in HepG2, HepG2/ADM and Huh7 cells. Then, we detected several key members of this pathway by western blotting. Consistent with β-catenin, Dvl-2, Dvl-3, and phospho-GSK-3β (Ser9) were depressed sharply after dalbinol treatment (Figure 3A). Additionally, there were reduced Cyclin D1 and c-Myc levels, two downstream effectors of Wnt/β-catenin signaling (Figure 3A).

Nuclear β-catenin accumulation has been shown to propel the development of many tumors. We next examined whether dalbinol exerted its anti-cancer effect by decreasing the accumulation of nuclear β-catenin. To this end, we made nuclear and cytoplasmic fractions and analyzed β-catenin levels in each. Both fractions showed reduced β-catenin levels following dalbinol treatment, though cytoplasmic β-catenin was reduced more significantly than nuclear β-catenin (Figure 3B). Immunofluorescence also suggested that dalbinol decreased nuclear and cytoplasmic β-catenin levels, especially in Huh7 cells (Figure 3C). Taken together, these experiments confirmed that dalbinol inhibited cell proliferation by downregulating nuclear and cytoplasmic β-catenin levels in HepG2, HepG2/ADM and Huh7 cells.

### Dalbinol blocked Wnt/β-catenin signaling by increasing β-catenin degradation

**Figure 4:**
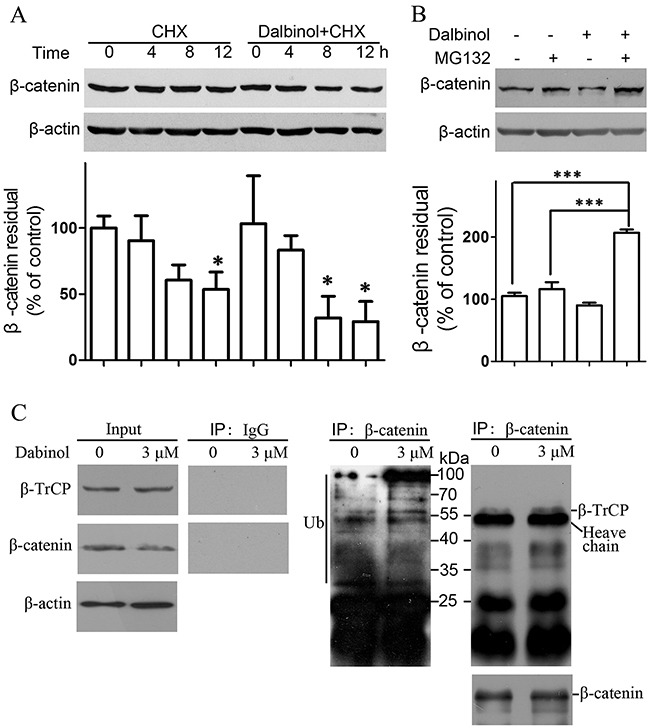
Dalbinol increased β-catenin degradation through the ubiquitin-proteasome pathway **(A)** Dalbinol shorten the half-life of β-catenin in HepG2/ADM cells treated with 5 μM dalbinol for 2 h followed by 25 μg/mL CHX for 12 h. **(B)** MG132 reversed the dalbinol-mediated β-catenin degradation in HepG2/ADM cells pretreated with 3 μM dalbinol for 2 h followed by 0.5 μM MG132 for 24 h. **(C)** The interaction between β-catenin and β-TrCP was increased along with an enhancement of ubiquitinated β-catenin levels in HepG2/ADM cells exposed to dalbinol at 3 μM for 24 h.

As shown in Figure 4B, cytoplasmic β-catenin was reduced more significantly than nuclear β-catenin, which implied that that dalbinol may promote the cytoplasmic degradation of β-catenin by decreasing Dvl-2/3 expression and increasing GSK-3β activity. To determine the underlying mechanism of the decreased expression of total β-catenin by dalbinol, we traced the half-life of β-catenin using cycloheximide (CHX) [38]. These results revealed that total β-catenin was reduced in a time-dependent manner (0, 4, 8, and 12 h) by CHX (25 μg/mL) alone in HepG2/ADM cells, while 2h pretreatment with 5 μM dalbinol accelerated the CHX-mediated reduction in total β-catenin (Figure 4A). In the same way, we monitored the stability of β-catenin with the proteasomal inhibitor MG132 in HepG2/ADM cells [38]. The data showed that MG132 reversed dalbinol-induced proteasomal β-catenin degradation (Figure 4B).

Furthermore, to test whether dalbinol promotes cytoplasmic β-catenin degradation, we employed a co-immunoprecipitation assay. This showed that the interaction between β-TrCP, an E3 ubiquitin ligase involved directly in β-catenin degradation, and β-catenin increased in HepG2/ADM cells treated with dalbinol (Figure 4C, right). This enhanced interaction between β-TrCP and β-catenin is thought to be an important event in β-catenin degradation. Moreover, we found increased levels of ubiquitinated-β-catenin, which suggested dalbinol enabled β-TrCP/β-catenin complex formation leading to β-TrCP-mediated β-catenin degradation (Figure 4C).

## DISCUSSION

Data from several laboratories have shown that rotenoids of different origins, such as deguelin, boeravinone and 6-deoxyclitoriacetal exert cytostatic effects on cancer cells derived from various tissues. However, dalbinol, another rotenoid derivative, has not been reported for its anti-cancer activity. In this study, we tested the anti-cancer activity of dalbinol in the HCC cell lines HepG2, HepG2/ADM and Huh7. Particularly, we uncovered that dalbinol exerted an anti-proliferative activity by promoting β-catenin degradation, thus blocking Wnt signaling. Our data suggested dalbinol possessed a lower cytotoxicity than rotenone in immortalized normal hepatocytes (Figure 1C). With regard to chemical structure, we speculate that the 12a-hydroxyl group causes the space conformation of dalbinol to differ from that of rotenone. Notably, although Adriamycin is a widely used first-line drug for various cancers, including HCC, drug resistance is still a major challenge that has impeded its clinical application. To our surprise, dalbinol had more inhibitory effects on HepG2/ADM not HepG2 and Huh7 cells than Adriamycin (Figure 1E), which suggests that dalbinol probably selectively overcome Adriamycin resistance.

Upon dalbinol challenge, apoptotic cell death was triggered. This was demonstrated by flow cytometry using an Annexin V probe; cleavage of pro-caspase-3 and PARP was also noted. Downregulation of survivin [39], Mcl-1 [40] and upregulation of Bax [41] and Bim [42] supported the notion that dalbinol induced apoptosis, at least partially.

As dysregulated Wnt/β-catenin signaling is frequently seen in HCC, it has been implicated in HCC development at the molecular level [43, 44]; these changes may accelerate hepatocyte proliferation leading to generation of premalignant clones, followed by the establishment of fully malignant disease after the acquisition of additional genetic lesions [45, 46]. To explore the mechanisms of β-catenin repression by dalbinol, we examined whether dalbinol contributed to β-catenin degradation that is mediated by the ubiquitin-proteasome system [47–49]. Our data suggested that β-catenin synthesis was under constant renewal, as shown by its decreased levels after adding CHX, an inhibitor of a *de novo* protein synthesis. Besides, dalbinol-induced decrease in the GSK-3β (pSer9) level meant GSK-3β was activated, which resulted in phosphorylation of β-catenin (Figure 3A). Once phosphorylated, β-catenin was specifically recognized and bound by β-TrCP E3 ubiquitin ligase to suffer degradation through the ubiquitin-proteasome system. The addition of MG132 (a proteasome inhibitor) remarkably blocked dalbinol-induced β-catenin degradation, which further supported the notion that dalbinol facilitated β-TrCP/β-catenin complex to decrease β-catenin levels by potentiating the activity of the ubiquitin-proteasome system (Figure 4B). Theoretically, when cells were treated with both dalbinol and MG132 for 24 hours, the β-catenin level would be more than, or equal to, or less than that of control. In our data, higher level of β-catenin was observed in cells exposed on both dalbinol and MG132 than control, which meant that MG132 vigorously reversed the degradation of β-catenin by dalbinol, also suggested that dalbinol reduced β-catenin level by ubiquitin-proteasome system.

**Figure 5:**
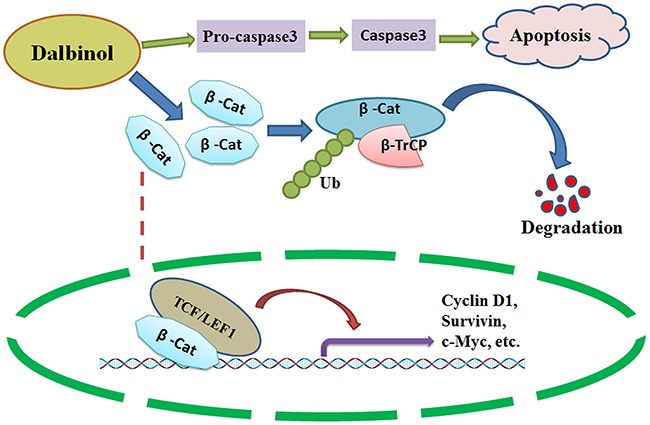
A schematic diagram of the possible anti-cancer mechanism of dalbinol in HCC Dalbinol exerted anti-cancer activity by promoting β-catenin degradation and decreasing nuclear β-catenin accumulation, it also partially induced apoptosis.

Taken together, our data indicate that dalbinol not only induced apoptosis in HepG2, HepG2/ADM and Huh7 cells, but also suppressed proliferation and promoted β-catenin degradation through engagement of the ubiquitin-proteasome pathway (Figure 5). Therefore, targeting β-catenin might be a promising therapeutic strategy for HCC treatment, and dalbinol might be a desirable candidate therapeutic compound that targets the Wnt/β-catenin pathway.

## MATERIALS AND METHODS

### Cell culture

The human HCC cell lines HepG2 and Huh7 were obtained from the Cell Bank of the Chinese Academy of Sciences (Shanghai, China). Adriamycin resistant HepG2/ADM cells were kindly provided by Dr. Caiguo Ye (Guangdong Medical University). HepG2, HepG2/ADM and Huh7 cells were cultured with DMEM (high glucose, Thermo Fisher Scientific, Waltham, MA, USA) with 10% fetal bovine serum (FBS, Gibco, Thermo Fisher Scientific) in a 5% CO_2_ humidified incubator at 37°C. Cells were split after trypsinization when they reached 70%–90% confluence.

### Chemicals and reagents

The natural compound dalbinol was extracted and purified from the seeds of *A fruticosa L*. by Dr. Xin Wu (Guangdong Medical University) using previously described procedures [26]. The *A fruticosa L*. seeds were deposited in the Guangdong Key Laboratory for Research and Development of Natural Drugs (Guangdong Medical University, Zhanjiang, China), and the voucher specimen number was 20130910. Rotenone and CHX were from MP Biomedicals Corporation (Santa Ana, CA, USA), propidium iodide was from Sigma-Aldrich (Shanghai, China), MG132 was from Calbiochem (San Diego, CA, USA) and DAPI was from Beyotime (Shanghai, China). Specific anti-Caspase-3 p17 (Cat: 25546-1-AP), Dvl-3 (Cat: 13444-1-AP), Cyclin D1 (Cat: 60186-1-lg) and IgG (Cat: B900610) antibodies were from Proteintech (Rosemont, IL, USA). Antibodies against Cleaved PARP (#5625), Survivin (#2808), Mcl-1 (#5453), Bim (#2933), Bax (#5023), Ubiquitin (#3936), β-catenin (#8480), GSK-3β (pSer9) (#9322), β-TrCP (#11984) and β-actin (#3700) were from Cell Signaling Technology (Danvers, MA, USA). Protein A/G PLUS-Agarose (sc-2003) and the antibody against Dvl-2 (sc-13974) were from Santa Cruz Biotechnology (Dallas, Texas, USA). Annexin V-FITC (Cat: 51-65874X) and antibodies against GSK-3β (Cat: 610201) and c-Myc (Cat: 551101) were obtained from BD Biosciences Pharmingen (San Jose, CA, USA). Histone 3 antibody (orb136531) was bought from Biorbyt (Cambridge, UK). Goat Anti-Rabbit IgG (H+L), DyLight (tm) 488 Conjugated (No. 35552) was from Pierce Biotechnology (Rockford, IL, USA).

### Cell viability assay

The Cell Counting Kit-8 (CCK-8) assay was used to evaluate the effect of dalbinol on cell viability. Briefly, 2×10^3^cells were seeded in each well of 96-well microplates and co-incubated with dalbinol at different concentrations for 72 h. Cell viability was evaluated as a percentage of the control cells, which were treated with equal volumes of DMSO (<0.1%) in DMEM. Three hours prior to culture termination, 10 μL of CCK-8 liquid was added to each well, and optical density was read on a 96-well microplate reader at 450 nm. IC_50_ values were calculated using GraphPad Prism 5.0 software (GraphPad Inc., La Jolla, CA, USA).

### Colony formation assay

For colony formation assays, 500 cells/well were seeded in a 12-well plate. After over-night incubation, the cells were exposed to concentration gradients of dalbinol and cultured for 9 days. The medium mixed with compound was replaced on the 4th day. On day 9, cells were washed with PBS, fixed in ice-cold methanol for 10 min, and stained with crystal violet for 15 min at room temperature. After rinsing with distilled water twice and drying at room temperature, images of the colonies were scanned using an HP scanner. Representative images were shown. Colonies were counted using an inverted phase-contrast microscope, and aggregates of ≥50 cells were identified as a colony. All numbers from three independent experiments were normalized to the average of controls using GraphPad Prism 5.0 software.

### Apoptosis assay

Apoptosis was detected by FACS after Annexin V/PI double staining. Cells were cultured in the presence of different concentrations of dalbinol for 24 h. Then they were pelleted by centrifugation, washed twice in cold PBS, then washed in 500 μL binding buffer (10 mM Hepes, pH 7.4; 140 mM NaCl; 2.5 mM CaCl_2_). Next, the cells were resuspended in 100 μL binding buffer containing 3% (v/v) Annexin V-FITC and incubated at room temperature for 20 min in the dark. After incubation, the cells were resuspended in 400 μL binding buffer. PI was added before FACS analysis. Apoptosis rates were analyzed with WinMDI 2.9 software (http://facs.scripps.edu/).

### Western blots

Total protein was extracted from cultured cells using RIPA lysis buffer, supplemented with freshly added 100 mM PMSF. The cytoplasmic and nuclear proteins were extracted using the Nuclear and Cytoplasmic Protein Extraction Kit (Beyotime) according to the manufacturer's instructions. The lysate collected after ultracentrifugation was quantitated with the BCA Protein Assay Kit, and total protein was separated by SDS-PAGE, then electro-blotted to nitrocellulose filters. The filters were blocked with 5% non-fat milk in PBS before probing with specific primary antibodies at different conditions. The filters were then washed with 1% Tween 20 in PBS, and conjugated with the appropriate secondary antibodies, and developed.

### Immunofluorescence staining

Cells cultured in 35 mm dishes with 14 mm glass bottom wells were washed with PBS twice, fixed for 15 min in PBS containing 4% paraformaldehyde, and permeabilized with 0.5% NP-40 and 1% BSA in PBS for 15 min. After washing with PBS for 5 min thrice and blocking with 5% BSA for 1 h, the cells were incubated with β-catenin primary antibody in a humidity chamber at 4°C overnight, followed by washing with PBS for 5 min thrice and incubation with DyLight (tm) 488-conjugated (green fluorescence representing β-catenin) secondary antibody together with DAPI at 4°C for 1 h in the dark. The cells were mounted onto slides and visualized using a Leica TCS SP5 II confocal laser-scanning microscope image system (Wetzlar, Germany).

### Co-Immunoprecipitation (co-IP)

For co-IPs, cells cultured on 60 mm dishes were washed with PBS and lysed with lysis buffer (50 mM Tris-HCl, pH7.4; 150 mM NaCl; 2 mM EDTA-Na_2_, 1% NP-40, 10% Glucose) on ice for 1 h. After centrifuging at 15,000 *×g* for 10 min at 4°C, supernatant was collected and 600 μg total protein in 800 μL lysis buffer was incubated with 6 μg primary antibody (w/w, antibody: total protein=1:100) at 4°C overnight with rocking (IgG control). Meanwhile, 50 μg total protein was saved and mixed with loading buffer as input for western blotting. For the co-IPs, 60 μL of prewashed protein A/G agarose beads were incubated with 600 μg samples at 4°C for 1 h with rocking. After incubation, the immune complexes were washed three times with lysis buffer, and proteins were eluted by boiling in loading buffer, followed by separation by 10% SDS-PAGE and western blot analysis using the corresponding antibodies.

### Statistical analysis

GraphPad Prism 5.0 software (GraphPad Inc.) was used to perform all statistical analyses. Student's t test was employed to compare data between two groups, and comparisons among multiple groups employed one-way ANOVA with Tukey's post hoc intergroup comparisons. *P* < 0.05 was considered statistically significant.

## Abbreviations

HCC: hepatocellular carcinoma
ADM: adriamycin
HCV: hepatitis C virus
APC: adenomatous polyposis coli
CHX: cycloheximide
PBS: phosphate-buffered saline
FBS: fetal bovine serum
PI: propidium iodide.

## References

[1] El-Serag HB, Mason AC (1999). Rising incidence of hepatocellular carcinoma in the United States. N Engl J Med.

[2] Siegel RL, Miller KD, Jemal A (2015). Cancer statistics, 2015. CA Cancer J Clin.

[3] El-Serag HB (2004). Hepatocellular carcinoma: recent trends in the United States. Gastroenterology.

[4] Parkin DM, Bray F, Ferlay J, Pisani P (2005). Global cancer statistics, 2002. CA Cancer J Clin.

[5] Logan CY, Nusse R (2004). The Wnt signaling pathway in development and disease. Annu Rev Cell Dev Biol.

[6] MacDonald BT, Tamai K, He X (2009). Wnt/beta-catenin signaling: components, mechanisms, and diseases. Dev Cell.

[7] Lee HC, Kim M, Wands JR (2006). Wnt/Frizzled signaling in hepatocellular carcinoma. Front Biosci.

[8] Lachenmayer A, Alsinet C, Savic R, Cabellos L, Toffanin S, Hoshida Y, Villanueva A, Minguez B, Newell P, Tsai HW, Barretina J, Thung S, Ward SC (2012). Wnt-pathway activation in two molecular classes of hepatocellular carcinoma and experimental modulation by sorafenib. Clin Cancer Res.

[9] Shi Q, Shi X, Zuo G, Xiong W, Li H, Guo P, Wang F, Chen Y, Li J, Chen DL (2016). Anticancer effect of 20(S)-ginsenoside Rh2 on HepG2 liver carcinoma cells: Activating GSK-3beta and degrading beta-catenin. Oncol Rep.

[10] Chen Y, Yu D, Zhang C, Shang B, He H, Chen J, Zhang H, Zhao W, Wang Z, Xu X, Zhen Y, Shao RG (2015). Lidamycin inhibits tumor initiating cells of hepatocellular carcinoma Huh7 through GSK3 β/β-catenin pathway. Mol Carcinog.

[11] Henderson BR, Fagotto F (2002). The ins and outs of APC and beta-catenin nuclear transport. EMBO Rep.

[12] Polakis P (2000). Wnt signaling and cancer. Genes Dev.

[13] Morin PJ (1999). β-catenin signaling and cancer. Bioessays.

[14] Satoh S, Daigo Y, Furukawa Y, Kato T, Miwa N, Nishiwaki T, Kawasoe T, Ishiguro H, Fujita M, Tokino T, Sasaki Y, Imaoka S, Murata M (2000). AXIN1 mutations in hepatocellular carcinomas, and growth suppression in cancer cells by virus-mediated transfer of AXIN1. Nat Genet.

[15] Henderson BR, Galea M, Schuechner S, Leung L (2002). Lymphoid enhancer factor-1 blocks adenomatous polyposis coli-mediated nuclear export and degradation of β-catenin. Regulation by histone deacetylase 1. J Biol Chem.

[16] Munemitsu S, Albert I, Souza B, Rubinfeld B, Polakis P (1995). Regulation of intracellular beta-catenin levels by the adenomatous polyposis coli (APC) tumor-suppressor protein. Proc Natl Acad Sci U S A.

[17] Kimelman D, Xu W (2006). β-catenin destruction complex: insights and questions from a structural perspective. Oncogene.

[18] Forner A, Llovet JM, Bruix J (2012). Hepatocellular carcinoma. Lancet.

[19] de Lope CR, Tremosini S, Forner A, Reig M, Bruix J (2012). Management of HCC. J Hepatol.

[20] Llovet JM, Ricci S, Mazzaferro V, Hilgard P, Gane E, Blanc JF, de Oliveira AC, Santoro A, Raoul JL, Forner A, Schwartz M, Porta C, Zeuzem S (2008). Sorafenib in advanced hepatocellular carcinoma. N Engl J Med.

[21] Wilhelm SM, Adnane L, Newell P, Villanueva A, Llovet JM, Lynch M (2008). Preclinical overview of sorafenib, a multikinase inhibitor that targets both Raf and VEGF and PDGF receptor tyrosine kinase signaling. Mol Cancer Ther.

[22] Zhu AX, Sahani DV, Duda DG, di Tomaso E, Ancukiewicz M, Catalano OA, Sindhwani V, Blaszkowsky LS, Yoon SS, Lahdenranta J, Bhargava P, Meyerhardt J, Clark JW (2009). Efficacy, safety, and potential biomarkers of sunitinib monotherapy in advanced hepatocellular carcinoma: a phase II study. J Clin Oncol.

[23] Faivre S, Raymond E, Boucher E, Douillard J, Lim HY, Kim JS, Zappa M, Lanzalone S, Lin X, Deprimo S, Harmon C, Ruiz-Garcia A, Lechuga MJ (2009). Safety and efficacy of sunitinib in patients with advanced hepatocellular carcinoma: an open-label, multicentre, phase II study. Lancet Oncol.

[24] Pokuri VK, Tomaszewski GM, Ait-Oudhia S, Groman A, Khushalani NI, Lugade AA, Thanavala Y, Ashton EA, Grande C, Fetterly GJ, Iyer R (2016). Efficacy, safety, and potential biomarkers of sunitinib and transarterial chemoembolization (TACE) combination in advanced hepatocellular carcinoma (HCC): phase II trial. Am J Clin Oncol.

[25] Thomas MB, Chadha R, Glover K, Wang X, Morris J, Brown T, Rashid A, Dancey J, Abbruzzese JL (2007). Phase 2 study of erlotinib in patients with unresectable hepatocellular carcinoma. Cancer.

[26] Kaseb AO, Morris JS, Iwasaki M, Al-Shamsi HO, Raghav KP, Girard L, Cheung S, Nguyen V, Elsayes KM, Xiao L, Abdel-Wahab R, Shalaby AS, Hassan M (2016). Phase II trial of bevacizumab and erlotinib as a second-line therapy for advanced hepatocellular carcinoma. Onco Targets Ther.

[27] Villanueva A, Minguez B, Forner A, Reig M, Llovet JM (2010). Hepatocellular carcinoma: novel molecular approaches for diagnosis, prognosis, and therapy. Annu Rev Med.

[28] Hoshida Y, Toffanin S, Lachenmayer A, Villanueva A, Minguez B, Llovet JM (2010). Molecular classification and novel targets in hepatocellular carcinoma: recent advancements. Semin Liver Dis.

[29] Suh YA, Kim JH, Sung MA, Boo HJ, Yun HJ, Lee SH, Lee HJ, Min HY, Suh YG, Kim KW, Lee HY (2013). A novel antitumor activity of deguelin targeting the insulin-like growth factor (IGF) receptor pathway via up-regulation of IGF-binding protein-3 expression in breast cancer. Cancer Lett.

[30] Mehta R, Katta H, Alimirah F, Patel R, Murillo G, Peng X, Muzzio M, Mehta RG (2013). Deguelin action involves c-Met and EGFR signaling pathways in triple negative breast cancer cells. PLoS One.

[31] Yang YL, Ji C, Bi ZG, Lu CC, Wang R, Gu B, Cheng L (2013). Deguelin induces both apoptosis and autophagy in cultured head and neck squamous cell carcinoma cells. PLoS One.

[32] Ahmed-Belkacem A, Macalou S, Borrelli F, Capasso R, Fattorusso E, Taglialatela-Scafati O, Di Pietro A (2007). Nonprenylated rotenoids, a new class of potent breast cancer resistance protein inhibitors. J Med Chem.

[33] Sangthong S, Krusong K, Ngamrojanavanich N, Vilaivan T, Puthong S, Chandchawan S, Muangsin N (2011). Synthesis of rotenoid derivatives with cytotoxic and topoisomerase II inhibitory activities. Bioorg Med Chem Lett.

[34] Wu X, Liao HB, Li GQ, Liu Y, Cui L, Wu KF, Zhu XH, Zeng XB (2015). Cytotoxic rotenoid glycosides from the seeds of Amorpha fruticosa. Fitoterapia.

[35] Kim YS, Ryu YB, Curtis-Long MJ, Yuk HJ, Cho JK, Kim JY, Kim KD, Lee WS, Park KH (2011). Flavanones and rotenoids from the roots of Amorpha fruticosa L. that inhibit bacterial neuraminidase. Food Chem Toxicol.

[36] Thamilselvan V, Menon M, Thamilselvan S (2011). Anticancer efficacy of deguelin in human prostate cancer cells targeting glycogen synthase kinase-3 beta/beta-catenin pathway. Int J Cancer.

[37] Murillo G, Peng X, Torres KE, Mehta RG (2009). Deguelin inhibits growth of breast cancer cells by modulating the expression of key members of the Wnt signaling pathway. Cancer Prev Res (Phila).

[38] Zhu X, Chen L, Jiang S, Chen C, Yao Y, Chen D, Xue H, Pan J (2014). PQJS380: a novel lead compound to induce apoptosis in acute lymphoblastic leukemia cells. Cancer Biol Ther.

[39] Ma J, Feng Y, Liu Y, Li X (2016). PUMA and survivin are involved in the apoptosis of HepG2 cells induced by microcystin-LR via mitochondria-mediated pathway. Chemosphere.

[40] Wu S, Liu F, Xie L, Peng Y, Lv X, Zhu Y, Zhang Z, He X (2015). miR-125b suppresses proliferation and invasion by targeting MCL1 in gastric cancer. Biomed Res Int.

[41] Wei MC, Zong WX, Cheng EH, Lindsten T, Panoutsakopoulou V, Ross AJ, Roth KA, MacGregor GR, Thompson CB, Korsmeyer SJ (2001). Proapoptotic BAX and BAK: a requisite gateway to mitochondrial dysfunction and death. Science.

[42] Weber A, Paschen SA, Heger K, Wilfling F, Frankenberg T, Bauerschmitt H, Seiffert BM, Kirschnek S, Wagner H, Hacker G (2007). BimS-induced apoptosis requires mitochondrial localization but not interaction with anti-apoptotic Bcl-2 proteins. J Cell Biol.

[43] Roberts LR, Gores GJ (2005). Hepatocellular carcinoma: molecular pathways and new therapeutic targets. Semin Liver Dis.

[44] Villanueva A, Newell P, Chiang DY, Friedman SL, Llovet JM (2007). Genomics and signaling pathways in hepatocellular carcinoma. Semin Liver Dis.

[45] Chen JD, Yang HI, Iloeje UH, You SL, Lu SN, Wang LY, Su J, Sun CA, Liaw YF, Chen CJ, Risk Evaluation of Viral Load Elevation and Associated Liver Disease/Cancer in HBV (REVEAL-HBV) Study Group (2010). Carriers of inactive hepatitis B virus are still at risk for hepatocellular carcinoma and liver-related death. Gastroenterology.

[46] Bruix J, Gores GJ, Mazzaferro V (2014). Hepatocellular carcinoma: clinical frontiers and perspectives. Gut.

[47] Aberle H, Bauer A, Stappert J, Kispert A, Kemler R (1997). beta-catenin is a target for the ubiquitin-proteasome pathway. EMBO J.

[48] Liu C, Kato Y, Zhang Z, Do VM, Yankner BA, He X (1999). β-Trcp couples β-catenin phosphorylation-degradation and regulates Xenopus axis formation. Proc Natl Acad Sci U S A.

[49] Gao C, Chen G, Romero G, Moschos S, Xu X, Hu J (2014). Induction of Gsk3beta-beta-TrCP interaction is required for late phase stabilization of beta-catenin in canonical Wnt signaling. J Biol Chem.

